# US County–Level Variation in Preterm Birth Rates, 2007-2019

**DOI:** 10.1001/jamanetworkopen.2023.46864

**Published:** 2023-12-08

**Authors:** Sadiya S. Khan, Adam S. Vaughan, Katharine Harrington, Laura Seegmiller, Xiaoning Huang, Lindsay R. Pool, Matthew M. Davis, Norrina B. Allen, Simon Capewell, Martin O’Flaherty, Gregory E. Miller, Roxana Mehran, Birgit Vogel, Kiarri N. Kershaw, Donald M. Lloyd-Jones, William A. Grobman

**Affiliations:** 1 Department of Medicine, Northwestern University Feinberg School of Medicine, Chicago, Illinois; 2 Department of Preventive Medicine, Northwestern University Feinberg School of Medicine, Chicago, Illinois; 3 Division for Heart Disease and Stroke Prevention, Centers for Disease Control and Prevention, Atlanta, Georgia; 4 Department of Pediatrics, Northwestern University Feinberg School of Medicine, Chicago, Illinois; 5 Institute of Population Health, University of Liverpool, Liverpool, United Kingdom; 6 Institute for Policy Research, Northwestern University, Evanston, Illinois; 7 Department of Psychology, Northwestern University, Evanston, Illinois; 8 Department of Medicine, Icahn School of Medicine at Mount Sinai, New York, New York; 9 Associate Editor, *JAMA Cardiology*; 10 Department of Obstetrics and Gynecology, The Ohio State University School of Medicine, Columbus

## Abstract

**Importance:**

Preterm birth is a leading cause of preventable neonatal morbidity and mortality. Preterm birth rates at the national level may mask important geographic variation in rates and trends at the county level.

**Objective:**

To estimate age-standardized preterm birth rates by US county from 2007 to 2019.

**Design, Setting, and Participants:**

This serial cross-sectional study used data from the National Center for Health Statistics composed of all live births in the US between 2007 and 2019. Data analyses were performed between March 22, 2022, and September 29, 2022.

**Main Outcomes and Measures:**

Age-standardized preterm birth (<37 weeks’ gestation) and secondarily early preterm birth (<34 weeks’ gestation) rates by county and year calculated with a validated small area estimation model (hierarchical bayesian spatiotemporal model) and percent change in preterm birth rates using log-linear regression models.

**Results:**

Between 2007 and 2019, there were 51 044 482 live births in 2383 counties. In 2007, the national age-standardized preterm birth rate was 12.6 (95% CI, 12.6-12.7) per 100 live births. Preterm birth rates varied significantly among counties, with an absolute difference between the 90th and 10th percentile counties of 6.4 (95% CI, 6.2-6.7). The gap between the highest and lowest counties for preterm births was 20.7 per 100 live births in 2007. Several counties in the Southeast consistently had the highest preterm birth rates compared with counties in California and New England, which had the lowest preterm birth rates. Although there was no statistically significant change in preterm birth rates between 2007 and 2019 at the national level (percent change, −5.0%; 95% CI, −10.7% to 0.9%), increases occurred in 15.4% (95% CI, 14.1%-16.9%) of counties. The absolute and relative geographic inequalities were similar across all maternal age groups. Higher quartile of the Social Vulnerability Index was associated with higher preterm birth rates (quartile 4 vs quartile 1 risk ratio, 1.34; 95% CI, 1.31-1.36), which persisted across the study period. Similar patterns were observed for early preterm birth rates.

**Conclusions and Relevance:**

In this serial cross-sectional study of county-level preterm and early preterm birth rates, substantial geographic disparities were observed, which were associated with place-based social disadvantage. Stability in aggregated rates of preterm birth at the national level masked increases in nearly 1 in 6 counties between 2007 and 2019.

## Key Points

**Question:**

What are the patterns and differences in preterm birth rates among US counties from 2007 to 2019?

**Findings:**

In this cross-sectional study, small area estimation models were applied to birth registration records from the National Center for Health Statistics and demonstrated increases in preterm birth rates in 15.4% of counties between 2007 and 2019. The absolute difference between the 90th and 10th percentile counties was stable during the study period with a gap of 6.4% in 2007 and 6.1% in 2019.

**Meaning:**

From 2007 to 2019, geographic disparities persisted, with increases in preterm birth rates in nearly 1 in 6 counties.

## Teaser

This cross-sectional study examines US county-level variation and trends in preterm birth rates from 2007 to 2019.

## Introduction

Preterm birth, defined as delivery at less than 37 weeks of gestation, is a leading cause of preventable neonatal morbidity and mortality in both the US and worldwide and represents one of the most common adverse pregnancy outcomes.^1,2,3^ Preterm birth imposes significant health and economic burdens, which include short- and long-term risks for both the offspring as well as the birthing individual.^4,5^ Emerging data highlight higher long-term risk of cardiovascular disease (CVD) in the birthing individual after delivering an infant preterm.^6,7,8^ Therefore, prevention of preterm birth has been highlighted as a national priority,^9^ including at the 2008 Surgeon General Conference on Preterm Birth,^10^ which emphasized the importance of surveillance programs to monitor preterm birth rates.^11^

The national US preterm birth rate increased modestly between 2014 and 2019 after a period of relative stability between 2007 and 2014.^2,12^ However, national trends may mask important local variation. A large body of research has documented significant county-level variation in a variety of other health outcomes (eg, life expectancy,^13^ CVD mortality,^14^ and COVID-19^15^). However, less is known about county-level rates, trends, and correlates of geographic variation in preterm birth. Although prior data are limited, studies have examined geographic variation in preterm birth rates at the county or state level,^16^ but these studies have not included trend analyses with contemporary data or applied small area models to generate robust and stable estimates, particularly in counties with small numbers of preterm births. Furthermore, the correlation of social vulnerability with patterns in preterm birth have been examined in health system data sets or only in a single calendar year.^17,18^ To examine the evolving epidemiology and county-level variation in preterm birth rates and trends, we leveraged nationwide data to (1) generate robust small area level estimates of preterm birth rates, (2) examine associations between county-level differences in social disadvantage and preterm birth rates, and (3) quantify trends from 2007 to 2019 in county-level preterm birth rates.

## Methods

### Data Sources

For this cross-sectional study, we obtained restricted birth registration data from the National Vital Statistics System of the National Center for Health Statistics (NCHS), which includes details on all live births in the US, including maternal age, gestational age at delivery, year, and county of residence from 2007 to 2019. Data on race and ethnicity were not included in this analysis given that this was a place-based and not an individual-level analysis. County-level data on social disadvantage were obtained from the Centers for Disease Control and Prevention with the Social Vulnerability Index (SVI).^19^ The institutional review board at Northwestern University determined this study was exempt from review and the need for informed consent due to the use of deidentified data. This study followed the Strengthening the Reporting of Observational Studies in Epidemiology (STROBE) reporting guideline.

### Analytic Sample

The primary outcome was preterm birth (<37 weeks of gestation), and the secondary outcome was early preterm birth (<34 weeks of gestation). Preterm, early preterm, and total live births were tabulated by county, 5-year maternal age groups between 15 and 44 years, and delivery year. The 5-year maternal age groupings were used consistently as recommended by the National Vital Statistics System for standard reporting. We excluded pregnant individuals younger than 15 years or 45 years or older given small numbers at the county level.

### Small Area Models

We used a bayesian multivariate space-time conditional autoregressive model to estimate annual county-level preterm and early preterm birth rates by age group. Additional detailed descriptions of the methods and the reliability of rates calculated are in the eMethods in Supplement 1. These models increase the precision of estimated rates, allowing the inclusion of counties and populations with small populations and small numbers of births.^20,21^ This model, whose details have been previously published, has been used extensively for estimating county-level CVD mortality rates to describe patterns and trends in county-level vital statistics.^20,22,23,24,25,26^ Briefly, this model is based on the Besag-York-Mollié conditional autoregressive model for spatially referenced count data.^27^ By iteratively estimating parameters within a Markov chain Monte Carlo algorithm and incorporating correlation across space, time, and age group, these models generate more precise rates, even in the presence of small case counts and populations.^17^ However, it is possible that the advantage of greater precision of rates with this modeling may make smaller differences in rates more difficult to observe. Specifically, temporal trends for each county will be smoother; thus, observing significant differences will require a larger magnitude of difference (ie, the temporal curve will be relatively flat due to the correlation structure). In addition, temporal trends in adjacent counties will require greater magnitude of differences to identify geographic disparities in nearby counties. These aspects of the modeling result in greater robustness, producing a more conservative approach to statistically significant differences when identified. Preterm birth rates were estimated as the medians of the posterior distributions defined by the Markov chain Monte Carlo iterations and were age standardized to the age distribution of individuals with live births in 2007, the first year included in the current analysis.

### Statistical Analysis

#### Association of Social Vulnerability With Preterm Birth Rates

County-level social disadvantage was represented by the SVI, which is composed of 4 themes: socioeconomic status, household composition and disability, minority status (which reflects the social constructs of race and ethnicity) and language, and housing and transportation.^19^ Themes are expressed as percentiles (range, 0-100), which are in turn summed and expressed as an overall percentile (range, 0-100), with a higher percentile indicating higher vulnerability. We classified counties based on quartile of the overall SVI and then used negative binomial regression to calculate rate ratios for estimated preterm and early preterm birth rates by SVI quartile for each year, with a time-varying SVI (using the 2010 SVI for years 2007-2011, the 2014 SVI for years 2012-2015, the 2016 SVI for years 2016-2017, and the 2018 SVI for years 2018-2019). Specifically, for county *i*, age group *k*, and year *t*, the model was *Y_ikt_* = *β_0ik_* *+* *β_1ik _SVI* *+* *ln(n_ikt_)*, where *Y* is the number of preterm births and *n* is the total number of births. The rate ratio was then calculated as *e^β1ik^*.

For 2019, we described the differences between the 10th and 90th percentile counties for the overall SVI and for each of the 4 themes. In secondary analyses, we also performed a series of ordinary least-squares regression models with the estimated rate as the dependent variable and SVI as the independent variable(s) to examine county-level variation in rates associated with SVI.

#### Trends in Preterm Birth Rates, 2007-2019

We measured temporal trends nationally and in each county by estimating percent change in preterm and early preterm birth rates using log-linear regression on estimated rates from 2007 to 2019. Separate regression models were run for each county using estimated rates for all years. Specifically, for county *i*, age group *k*, and year *t*, the model was *ln*(*λ_ikt_*) = *β_0ik_* + *β_1ik_*·*t*. The total percent change was then calculated as *100*(*e^12β^1ik* – *1*). Using the estimated percent change, we calculated the percentage of counties with increasing birth rates for each age group.

#### National, State, and CSA Rates and Trends

To further examine geographic patterns, we calculated rates at the national, state, and combined statistical area (CSA) levels by aggregating crude, unsuppressed county-level data. As defined by the US Census Bureau, CSAs are combinations of metropolitan and micropolitan census areas that have economic and social linkages; CSAs may cross state lines. Counties within each state that were not included in a CSA were also aggregated. Age-specific and age-standardized rates were then calculated using these crude values. The corresponding trends were then calculated as defined earlier. No additional analyses were performed for these national-, state-, and CSA-level results.

#### Secondary and Sensitivity Analysis

We performed secondary analyses to calculate age-specific preterm birth rates for women aged 20 to 44 years in 5-year intervals in each county with available data on more than 100 births in each age range given age-specific risks of preterm birth. The primary analysis included all pregnant people, because each birth has the potential to be preterm. However, given the deidentified nature of the National Vital Statistics System data set, multiple live births to the same pregnant individual could be included, so we performed a sensitivity analysis restricted to nulliparous individuals with a singleton live birth. All analyses were conducted using R software, version 4.2.1 (R Foundation for Statistical Computing). Data analyses were performed between March 22, 2022, and September 29, 2022.

## Results

Between 2007 and 2019, there were 51 044 482 live births in 2383 counties included. In 2007, the national age-standardized preterm birth rate was 12.6 (95% CI, 12.6-12.7) per 100 live births, and the early preterm birth rate was 3.6 (95% CI, 3.6-3.6) (Table 1). There were geographic differences in 2007 that persisted through 2019 at several geographic area levels, including state, CSA, and county levels (eFigures 1-4 in Supplement 1). Counties at the 90th percentile had preterm birth rates that were 60% higher than those that were in the 10th percentile throughout the study period; this difference across percentiles translated into 6.4 (95% CI, 6.2-6.7) more preterm births and 2.2 (95% CI, 2.1-2.3) more early preterm births per 100 live births. The gaps between the highest and lowest counties were 20.7 for preterm births and 7.2 for early preterm births per 100 live births in 2007; these differences were relatively unchanged in 2019.

**Table 1.** National Age-Standardized Rates of Preterm Birth Outcomes and Distribution of Age-Standardized Rates of Preterm Birth Among US Counties in 2007 and 2019

Year	No. of counties	National rates (per 100 live births)		US county-level age-standardized rates (per 100 live births)						
		Total No. of births	Age-standardized rate (95% CI)	Minimum	10th Percentile	Median (IQR)	90th Percentile	Maximum	90th-10th Percentile (95% CI)	90th-10th percentile ratio (95% CI)
**Preterm birth (<37 weeks’ gestation)**										
2007	2383	543 299	12.6 (12.6-12.7)	7.4	10.2	12.6 (11.2-14.6)	16.6	28.1	6.4 (6.2-6.7)	1.6 (1.6-1.7)
2019	2383	452 499	12.2 (12.1-12.2)	6.6	9.9	12.2 (10.8-13.9)	16.0	61.4	6.1 (5.8-6.4)	1.6 (1.6-1.7)
**Early preterm (<34 weeks’ gestation)**										
2007	2380	155 809	3.6 (3.6-3.6)	1.7	2.7	3.5 (3.1-4.1,)	4.9	8.9	2.2 (2.1-2.3)	1.8 (1.8-1.9)
2019	2380	129 767	3.5 (3.5-3.6)	2.0	2.7	3.5 (3.0-4.1)	4.9	21.9	2.2 (2.1-2.3)	1.8 (1.8-1.9)

**Figure 1.**
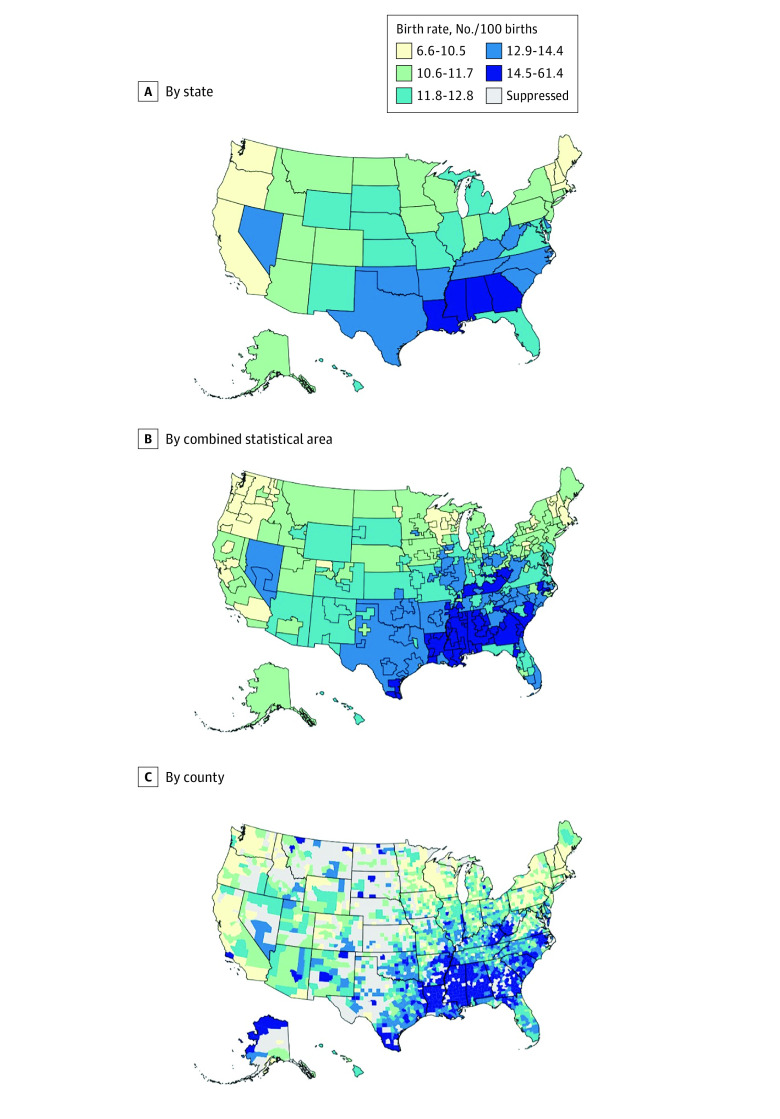
Age-Standardized Preterm Birth Rates in 2019 Among US States, Combined Statistical Areas, and Counties, for Those Aged 15 to 44 Years Age-standardized rates of preterm (<37 weeks’ gestation) are displayed across varying geographic scales in the US, with darker blue representing higher rates. For a county to be included on these maps, the estimated rates were required to be reliable (ie, the credible interval width was less than the point estimate) and the total number of births was 100 or greater for every year in the study period.

Several counties in the Southeast US consistently had the highest preterm birth rates (Figure 1). In contrast, counties in California and New England had the lowest preterm birth rates. Geographic patterns were similar for early preterm birth rates. In 2007, age-standardized preterm birth rates were greater in each higher quartile of SVI compared with the first quartile (2007 rate ratios for quartile 2 vs 1: 1.08 [95% CI, 1.06-1.10]; quartile 3 vs 1: 1.18 [95% CI, 1.15-1.20]; quartile 4 vs 1: 1.34 [95% CI, 1.31-1.36]). The rate ratios for SVI quartiles and preterm birth rates did not change from 2007 to 2019 (eFigure 5 in Supplement 1). When examining the SVI by theme, counties in the 10th and 90th percentile for preterm birth rates in 2019 had better (24th percentile) and worse (88th percentile) socioeconomic status, respectively, with similar adverse patterns for household composition and disability themes (Table 2). In secondary analyses, the socioeconomic status theme of SVI explained the largest county-level variation in preterm birth rates with an *R*^2^ of 0.27 (eTable 1 in Supplement 1). The magnitude of the coefficient for socioeconomic status was not attenuated even in sequential models adjusting for the other themes. In contrast, although each theme was associated with preterm birth rates in univariate analyses, coefficients for household composition and disability, minority status and language, and housing and transportation were attenuated in the fully adjusted model. The relative association between SVI and early preterm birth was similar to that for preterm birth (eTable 2 in Supplement 1).

**Table 2.** County-Level SVI Parameters in the 10th Percentile and 90th Percentile Counties for Preterm Birth Rate in the US in 2019

Parameter	US counties at ≤10th percentile for preterm birth rate (n = 258)		US counties at ≥90th percentile for preterm birth rate (n = 244)	
	Median value	Median percentile	Median value	Median percentile
Total SVI	6.1	0.27	9.8	0.87
**Theme 1: socioeconomic status**				
Overall SVI (theme 1)	1.1	0.24	3.3	0.88
Residents living below poverty level, %	10.9	0.24	23.3	0.88
Residents unemployed, %	4.6	0.35	8.0	0.83
Less than high school education, %	8.2	0.21	19.1	0.82
Median income, $	29 886	0.26	20 936	0.86
**Theme 2: household composition and disability**				
Overall SVI (theme 2)	1.6	0.21	2.4	0.80
Age ≥65 y, %	17.9	0.48	16.4	0.33
Age ≤17 y, %	21.0	0.32	23.4	0.64
Age ≥5 y with a disability, %	12.8	0.24	17.9	0.70
Single-parent household, %	7.0	0.30	10.9	0.85
**Theme 3: minority status and language**				
Overall SVI (theme 3)	0.9	0.43	1.2	0.62
Minority, %	10.0	0.35	42.8	0.83
Speak English “less than well,” %	0.8	0.51	0.5	0.36
**Theme 4: housing and transportation**				
Overall SVI (theme 4)	2.5	0.49	3.1	0.79
Multiunit structure, %	5.2	0.71	1.7	0.32
Mobile homes, %	6.5	0.31	20.6	0.80
Crowding, %	1.7	0.41	2.4	0.63
No vehicle, %	5.4	0.44	8.7	0.84
Group quarters, %	2.0	0.50	2.3	0.56

Abbreviation: SVI, Social Vulnerability Index.

**Figure 2.**
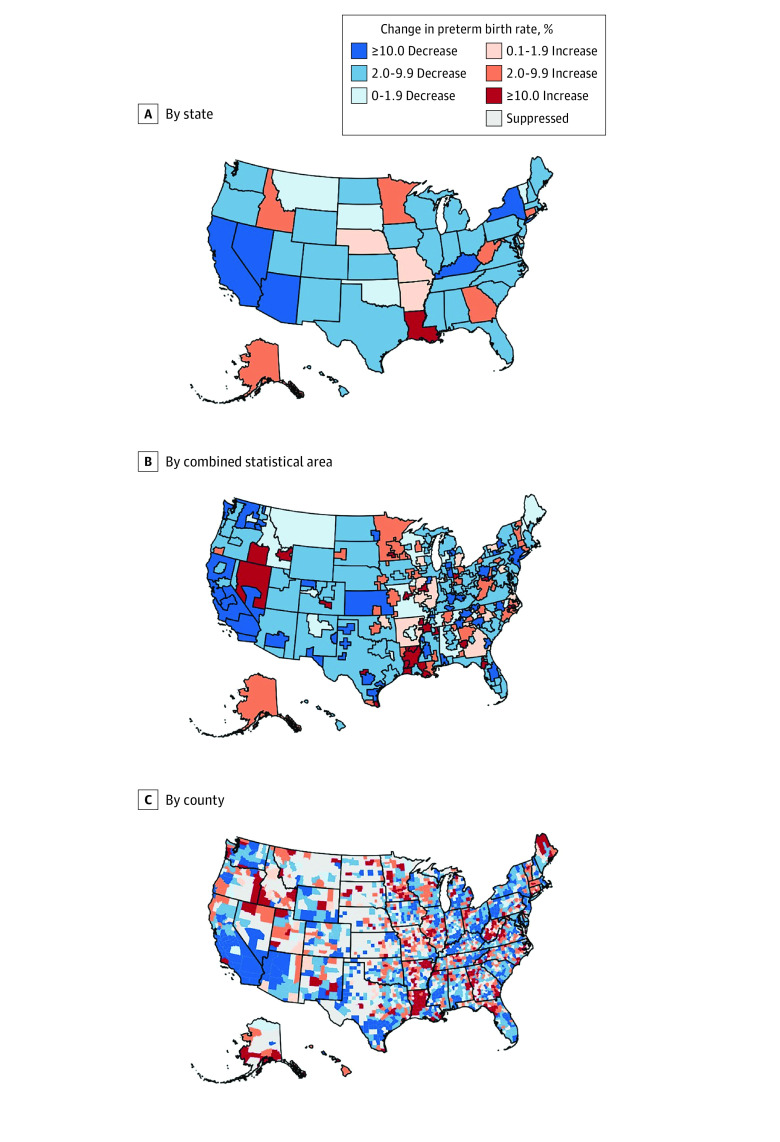
Total Percent Change in Age-Standardized Preterm Birth Rates Between 2007 and 2019 Among US States, Combined Statistical Areas, and Counties, for Those Aged 15 to 44 Years County-level percent change is displayed for preterm births (<37 weeks’ gestation), with darker red representing greater increases and darker blue representing greater decreases between 2007 and 2019 in the US. Mapped values are the point estimates of percent change calculated using log-linear regression from estimated rates. For a county to be included on these maps, the estimated rates were required to be reliable (ie, the credible interval width was less than the point estimate) and the total number of births was 100 or greater for every year in the study period.

**Figure 3.**
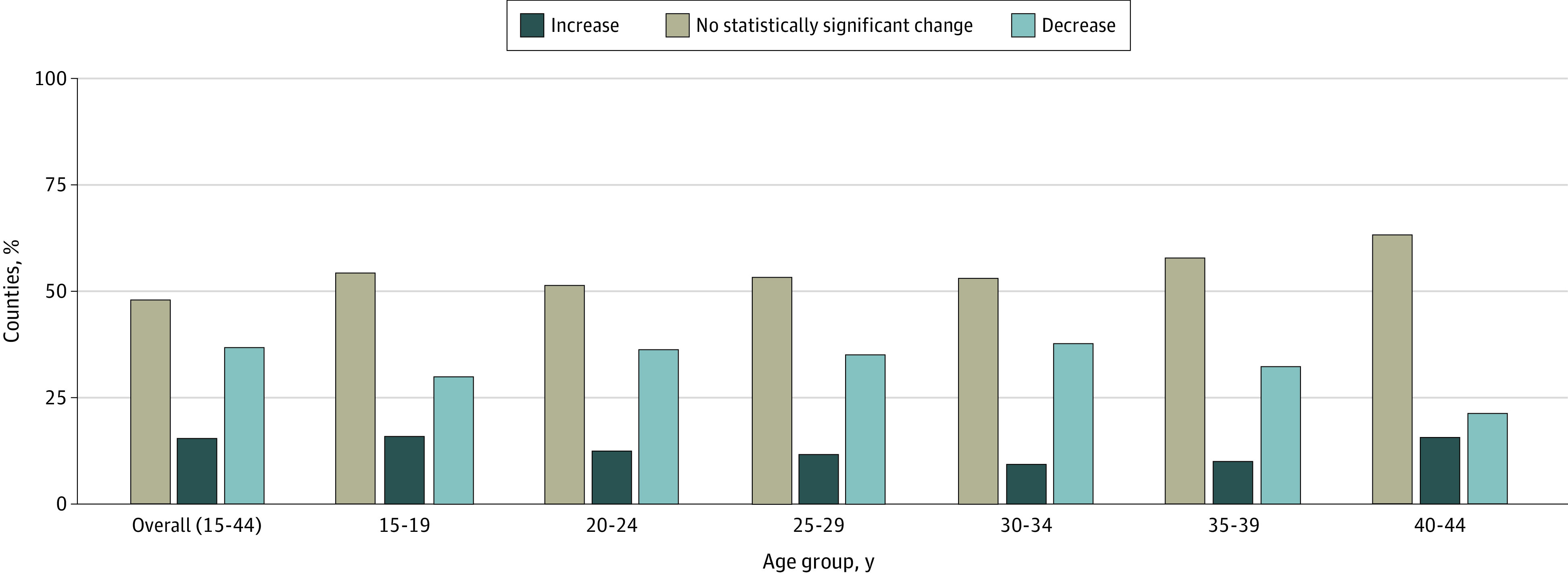
Distributions of Trends Among US Counties for Preterm Birth Between 2007 and 2019 Overall and Stratified by 5-Year Age Groups The proportion of counties with varying magnitudes of increases and decreases are displayed for the overall sample as well as in each 5-year age stratum. Statistically significant increases were calculated using log-linear regression based on 95% CIs that did not overlap with 0.

Between 2007 and 2019, the national preterm birth rate did not change significantly (total percent change, −5.0%; 95% CI, −10.7% to 0.9%) (eTable 3 in Supplement 1). Similarly, the national rate of early preterm birth did not change significantly (−3.0; 95% CI, −6.4 to 0.5). Stability in national trends (eFigure 6 in Supplement 1) obscured considerable variation in total percent change in preterm birth rates at the county level (Figure 2). For preterm birth, 15.4% (95% CI, 14.1%-16.9%) of counties experienced a statistically significant increase in their rates (Figure 3), with the greatest increases occurring in the upper Midwest. A larger proportion of counties had a statistically significant increase in early preterm birth rates (28.3%; 95% CI, 26.5%-30.1%) (eFigure 7 in Supplement 1).

Preterm birth rates had a U-shaped curve, with higher rates in those aged 15 to 19 years and in those aged 30 to 44 years throughout the study period. In 2019, 15- and 19-year-olds individuals had a median preterm birth rate of 13.7, and 40- to 44-year-old individuals had a median rate of 16.0 per 100 live births (eTable 4 in Supplement 1). Similar patterns were observed for early preterm birth rates. The absolute and relative geographic inequalities detailed here were similar across all maternal age groups and in the subset of nulliparous individuals with a singleton first live birth (eTable 5 in Supplement 1) for both preterm birth and early preterm birth rates.

## Discussion

This nationwide study of county-level preterm birth rates highlights 4 key findings. First, substantial county-level geographic disparities in preterm and early preterm birth rates were observed across the US. Second, counties with the greatest social disadvantage had the highest preterm and early preterm birth rates, which was predominantly explained by place-based socioeconomic factors. Third, the national trends in age-standardized preterm and early preterm birth rates, which were stable or decreased during the study period, obscured considerable variation in county-level trends, with approximately 15% of all counties having preterm birth rates that increased. Fourth, although absolute rates differed by age strata, relative patterns and trends for geographic differences were similar in each age group. These findings highlight the importance of quantifying trends in age-standardized and age-specific rates at the local level and that the detailed reporting of county-level patterns is a necessary first step to guide evidence-informed programs and policies at the local level to improve birth outcomes and equity.

Data on preterm birth rates have long been available at the national level, are a key indicator of population-level health, and have been widely used for public health planning. Reducing the preterm birth rate represents one of the key goals of Healthy People 2030,^28^ and proposed approaches have included improved health care access and quality, identification of and interventions for pregnant individuals at risk for preterm birth and related adverse pregnancy outcomes where delivery is an indicated treatment even if preterm (eg, hypertensive disorders of pregnancy), and health behavior counseling (eg, smoking cessation).^29^ However, monitoring of reliable county-level estimates of rates is needed to inform the design, implementation, and evaluation of community-based interventions. The robust small area models presented here may also provide an opportunity for evaluating and comparing effects of policies implemented in some, but not all, localities (eg, smoke-free legislation).^30^ Additionally, obstetricians, midwives, community workers, and programs, such as federally funded home visiting programs, may use these estimates to increase awareness, communicate the risks of preterm birth in the populations in which they work, and inform local strategies for prevention.

The present analysis demonstrated consistent and graded associations of preterm birth rates with county-level social disadvantage. Although all 4 themes that comprise the SVI were associated with preterm birth, county-level differences in preterm birth rates were predominantly explained by differences in the socioeconomic theme, which includes measures of income, employment, and education. The SVI thus provides potentially valuable information to contextualize variation among counties and to identify structural and policy-level opportunities for prevention of preterm birth, particularly among prevalent and emerging geographic “hot spots.” Beyond individual-level risk factors (eg, maternal age and smoking), there is a large and increasing body of evidence for the importance of broader place-based contextual factors on adverse pregnancy outcomes.^31^ A previous study found relationships between neighborhood-level characteristics and birth outcomes that persist after adjustment for individual-level factors.^31^ Hypothesized mechanisms through which local environments may influence birth outcomes include structural racism and its consequent ramifications, including environmental stress (eg, crime and police violence), availability of resources (eg, health care, food, and education), and the built environment (eg, housing conditions, air pollution, and other toxins).^32,33^ Moreover, the geographic patterns in preterm birth mirror those observed for CVD,^4,34^ which not only shares upstream social drivers of health but also is more likely after preterm birth. This finding demonstrates how multilevel interventions at the community level that financially invest in disadvantaged communities and address systemic barriers have the potential to improve outcomes across the life course. The SVI, particularly the socioeconomic theme or component, may therefore be a useful tool to implement to identify vulnerable communities and pregnant individuals who are at highest risk of preterm birth.

### Strengths and Limitations

This study has several strengths. First, the use of innovative modeling with well-validated small area models allows for more precise estimation and ranking of county-level rates in preterm births. Second, this study examined both preterm and early preterm births. Third, this study considered both age-standardized and age-specific preterm birth rates.

This study also has several limitations. First, there is the potential for miscoding of gestational age in birth records. However, high concordance between gestational age reported on the birth record and the medical record has been reported, and the NCHS represents the most robust national data source available for the surveillance of preterm births in the US.^35^ Second, the use of small area models may, in some cases, lead to oversmoothing; in addition, exclusion of the counties with the lowest number of births, which are more likely to be rural and have greater social disadvantage, may result in underestimation of relative differences across counties. In addition, there is the potential of bias toward recording urban residences in the data set, which may lead to further underestimation of rural-urban disparities. However, the use of small area models provides the most robust and stable estimates for modeling at the geographic scale when small numbers may be present as has been used in other publications for vital statistics.^36,37,38^ Third, the rates presented may underestimate overall rates of preterm birth because of the focus on birthing individuals aged 15 to 44 years, which was necessary given the small sample sizes for extreme ages. In addition, the aggregate rates and trends presented here differ from those published by the NCHS^39^ because we used age standardization to account for spatial and temporal changes in the age of birthing individuals. Fourth, the primary analysis uses deidentified data and therefore cannot exclude repeated individuals in the primary analysis. However, sensitivity analyses limited to nulliparous individuals resulted in the same patterns as the primary analysis. Fifth, this analysis used a composite score, the SVI, to represent county-level disadvantage, which may not fully represent social disadvantage at a smaller geographic scale (census tract) or at the individual level, which may have led to an underestimation of the importance of social factors in county-level patterns in preterm birth. Specifically, analysis at the county level may still mask significant heterogeneity within counties: census tract or neighborhood-level factors, such as residential segregation, which are well-established drivers of health inequities, might merit analysis in future studies. However, county-level SVI has been widely used in many other health outcomes as a proxy for social disadvantage and represents an important geographic scale that is relevant for policy makers.^40,41^ Sixth, the present data set does not differentiate between medically indicated and spontaneous preterm births, which limits the ability to examine how changes in clinical practice over time may influence preterm birth rates (eg, higher rates of medically indicated preterm birth due to hypertensive disorders of pregnancy). However, the aggregate of both medically indicated and spontaneous preterm birth rates is an important metric to monitor and modify.

## Conclusions

In this nationwide cross-sectional analysis, substantial variations in preterm birth rates were observed among US counties. These differences were associated with county-level variation in social vulnerability. Stability of age-adjusted preterm birth rates at the national level masked significant increases in nearly 1 in 6 US counties between 2007 and 2019. Policy makers, researchers, and clinicians may use these county-level prevalence and trend estimates to inform the development and evaluation of interventions to reduce place-based disparities in preterm birth and thus improve health for both the birthing individual and offspring.

## Notes

Supplement 1. **eMethods.** Supplemental Methods **eTable 1.** Multivariate Regression Results for the Association of County-Level Socioeconomic Characteristics (Social Vulnerability Index) and Preterm Birth Rates in the US in 2019 **eTable 2.** County-Level Socioeconomic Characteristics (Social Vulnerability Index) of the 10th Percentile and 90th Percentile Counties for Early Preterm Birth Rate in the US in 2019 **eTable 3.** Distribution of Percent Change in Age-Standardized and Age-Specific National and County-Level Preterm and Early Preterm Birth Rates From 2007 to 2019 in the US **eTable 4.** Age-Specific Rates of Preterm and Early Preterm Birth at the National and County-Level in the United States, 2019 **eTable 5.** National Age-Standardized Rates of Preterm Birth Outcomes and Distribution of Age-Standardized Rates of Preterm Birth Among US Counties in 2007 and 2019 Among Individuals With a Singleton First Live Birth **eFigure 1.** Age-Standardized Preterm Birth Rates in 2007 Among US States, Combined Statistical Areas, and Counties **eFigure 2.** Age-Standardized Early Preterm Birth Rates in 2007 Among US States, Combined Statistical Areas, and Counties **eFigure 3.** Age-Standardized Early Preterm Birth Rates in 2019 Among US States, Combined Statistical Areas, and Counties **eFigure 4.** Total Percent Change in Age-Standardized Early Preterm Birth Rates Between 2007-2019 Among US States, Combined Statistical Areas, and Counties **eFigure 5.** Annual Rate Ratios (95% CI) for Preterm Birth Rate and Early Preterm Birth Rate Stratified by Quartiles of the County-Level Social Vulnerability Index, 2007-2019 **eFigure 6.** Annual National and Distribution of County-Level Age-Standardized Preterm (A) and Early Preterm (B) Birth Rates **eFigure 7.** Distributions of Trends Among US Counties for Early Preterm Birth Between 2007-2019 Overall and Stratified by 5-Year Age Groups

Supplement 2.
Data Sharing Statement
