# Supplementary material for: US County–Level Variation in Preterm Birth Rates, 2007-2019
Source: JAMA Netw Open. 2023 Dec 8;6(12):e2346864. doi: 10.1001/jamanetworkopen.2023.46864 (PMC10709777; doi:10.1001/jamanetworkopen.2023.46864)
Supplement: Supplement 1. — eMethods. Supplemental Methods eTable 1. Multivariate Regression Results for the Association of County-Level Socioeconomic Characteristics (Social Vulnerability Index) and Preterm Birth Rates in the US in 2019 eTable 2. County-Level Socioeconomic Characteristics (Social Vulnerability Index) of the 10th Percentile and 90th Percentile Counties for Early Preterm Birth Rate in the US in 2019 eTable 3. Distribution of Percent Change in Age-Standardized and Age-Specific National and County-Level Preterm and Early Preterm Birth Rates From 2007 to 2019 in the US eTable 4. Age-Specific Rates of Preterm and Early Preterm Birth at the National and County-Level in the United States, 2019 eTable 5. National Age-Standardized Rates of Preterm Birth Outcomes and Distribution of Age-Standardized Rates of Preterm Birth Among US Counties in 2007 and 2019 Among Individuals With a Singleton First Live Birth eFigure 1. Age-Standardized Preterm Birth Rates in 2007 Among US States, Combined Statistical Areas, and Counties eFigure 2. Age-Standardized Early Preterm Birth Rates in 2007 Among US States, Combined Statistical Areas, and Counties eFigure 3. Age-Standardized Early Preterm Birth Rates in 2019 Among US States, Combined Statistical Areas, and Counties eFigure 4. Total Percent Change in Age-Standardized Early Preterm Birth Rates Between 2007-2019 Among US States, Combined Statistical Areas, and Counties eFigure 5. Annual Rate Ratios (95% CI) for Preterm Birth Rate and Early Preterm Birth Rate Stratified by Quartiles of the County-Level Social Vulnerability Index, 2007-2019 eFigure 6. Annual National and Distribution of County-Level Age-Standardized Preterm (A) and Early Preterm (B) Birth Rates eFigure 7. Distributions of Trends Among US Counties for Early Preterm Birth Between 2007-2019 Overall and Stratified by 5-Year Age Groups [file jamanetwopen-e2346864-s001.pdf]

Supplementary Online Content

Khan SS, Vaughan AS, Harrington K, et al. US county–level variation in preterm birth rates, 2007-2019. *JAMA Netw Open*. 2023;6(12):e2346864. doi:10.1001/jamanetworkopen.2023.46864

eMethods. Supplemental Methods
eTable 1. Multivariate Regression Results for the Association of County-Level Socioeconomic Characteristics (Social Vulnerability Index) and Preterm Birth Rates in the US in 2019
eTable 2. County-Level Socioeconomic Characteristics (Social Vulnerability Index) of the 10th Percentile and 90th Percentile Counties for Early Preterm Birth Rate in the US in 2019
eTable 3. Distribution of Percent Change in Age-Standardized and Age-Specific National and County-Level Preterm and Early Preterm Birth Rates From 2007 to 2019 in the US
eTable 4. Age-Specific Rates of Preterm and Early Preterm Birth at the National and County-Level in the United States, 2019
eTable 5. National Age-Standardized Rates of Preterm Birth Outcomes and Distribution of Age-Standardized Rates of Preterm Birth Among US Counties in 2007 and 2019 among Individuals with a Singleton First Live Birth
eFigure 1. Age-Standardized Preterm Birth Rates in 2007 Among US States, Combined Statistical Areas, and Counties*
eFigure 2. Age-Standardized Early Preterm Birth Rates in 2007 Among US States, Combined Statistical Areas, and Counties*
eFigure 3. Age-Standardized Early Preterm Birth Rates in 2019 Among US States, Combined Statistical Areas, and Counties
eFigure 4. Total Percent Change in Age-Standardized Early Preterm Birth Rates Between 2007-2019 among US States, Combined Statistical Areas, and Counties*
eFigure 5. Annual Rate Ratios (95% CI) for Preterm Birth Rate and Early Preterm Birth Rate Stratified by Quartiles of the County-Level Social Vulnerability Index, 2007-2019*
eFigure 6. Annual National and Distribution of County-Level Age-Standardized Preterm (A) and Early Preterm (B) Birth Rates
eFigure 7. Distributions of Trends* Among US Counties for Early Preterm Birth Between 2007-2019 Overall and Stratified By 5-Year Age Groups

This supplementary material has been provided by the authors to give readers additional information about their work.

© 2023 Khan et al. *JAMA Network Open.*

eMethods. Supplemental Methods

Small Area Models

We modeled Y_{ikt}, the number of preterm or early preterm births in county *i* and age group *k* during year *t* from a total number of births (n_{ikt}), using a Poisson distribution of the form $Y_{ikt} \sim Binom(n_{ikt}, \pi_{ikt})$, where π_{ikt} is the proportion of births that were preterm. To model π_{ikt}, we assume $logit(\pi_{ikt}) \sim N\left(\beta_{kt} + Z_{ikt}, \tau_k^2\right)$, where $logit(\pi_{ikt}) = log\left(\frac{\pi_{ikt}}{1-\pi_{ikt}}\right)$, β_{kt} is a random intercept for each group for each year with a vague normal distribution prior with mean zero and a variance of 100, Z_{ikt} is a spatiotemporal random effect that incorporates correlation between age groups, and τ_k^2 is a variance parameter with a weakly informative gamma prior.[1] Our models account for correlation across time, space, and age group by modeling the spatiotemporal random effect (Z_{ikt}) using the multivariate space-time conditional autoregressive (MSTCAR) model[13] based on the multivariate CAR model of Gelfand and Vounatsou.[2] Spatial correlation of the random effect for each county is defined by queen contiguity, which is a method of specifying the adjacency of counties commonly used in these types of analyses. Similarly, temporal correlation uses an approach similar to a standard autoregressive order 1 (AR(1)) model with a beta prior. Finally, correlations between age groups are estimated via an unstructured covariance matrix with an inverse Wishart prior.[1] We ran the MCMC algorithm with four chains for 6000 iterations, diagnosing convergence via trace plots for many model parameters and discarding the first 3000 iterations as burn-in. We generated estimates based on posterior medians, and 95% credible intervals were obtained by taking the 2.5- and 97.5-percentiles from the thinned post-burn-in samples.

Reliability of estimated rates

With these estimated rates, we then applied inclusion criteria to all 3,142 counties to ensure reporting of estimated reliable rates only for sufficiently large numbers of births and that, for each outcome, each maternal age group used a common set of counties for the entire study period. For a given maternal age group within a given county to be included in this analysis, we required that the estimated rates were statistically reliable (i.e., the credible interval width was less than the point estimate) and that the group-specific number of births was ≥100 for every year in the study period. For age-standardized rates, a given county's age-standardized rate was required to be statistically reliable and the total number of births was ≥100 for every year.

eReferences

1. Waller LA, Carlin BP, Xia H, Gelfand AE. Hierarchical spatio-temporal mapping of disease rates. Journal of the American Statistical association 1997;92(438):607-617.
2. Gelfand AE, Vounatsou P. Proper multivariate conditional autoregressive models for spatial data analysis. Biostatistics 2003;4(1):11-15.

eTable 1. Multivariate Regression Results for the Association of County-Level Socioeconomic Characteristics (Social Vulnerability Index) and Preterm Birth Rates in the US in 2019

	Model 1A[a]	Model 1B[a]	Model 1C[a]	Model 1D[a]	Model 2[b]	Model 3[c]	Model 4[d]
**Intercept**, coefficient (95% CI)	9.72 (9.5, 9.94)	10.62 (10.39, 10.85)	11.91 (11.65, 12.18)	11.09 (10.82, 11.36)	9.61 (9.38, 9.85)	9.24 (8.94, 9.54)	9.30 (8.99, 9.61)
**SVI Theme 1** (Socioeconomic Status), coefficient (95% CI)	5.80 (5.42, 6.18)				5.27 (4.76, 5.78)	5.05 (4.53, 5.57)	5.23 (4.63, 5.82)
**SVI Theme 2** (Household Composition & Disability), coefficient (95% CI)		4.19 (3.79, 4.59)			0.76 (0.26, 1.25)	0.92 (0.42, 1.42)	0.87 (0.36, 1.38)
**SVI Theme 3** (Minority Status and Language), coefficient (95% CI)			1.48 (1.04, 1.93)			0.76 (0.37, 1.15)	0.85 (0.43, 1.26)
**SVI Theme 4** (Housing and Transportation), coefficient (95% CI)				2.97 (2.53, 3.41)			-0.30 (-0.79, 0.19)
**R^2**	0.27	0.15	0.02	0.07	0.28	0.28	0.28
**Adjusted R^2**	0.27	0.15	0.02	0.07	0.28	0.28	0.28

[a]Model 1 represents each individual theme in a separate regression model
[b]Model 2 adjusts for SVI themes 1-2;
[c]Model 3: SVI Themes 1-3;
[d]Model 4: SVI Themes 1-4;

© 2023 Khan et al. *JAMA Network Open.*

eTable 2. County-Level Socioeconomic Characteristics (Social Vulnerability Index) of the 10th Percentile and 90th Percentile Counties for Early Preterm Birth Rate in the US in 2019

	**US Counties, ≤10th percentile for early preterm birth rate (N=231)**		**US Counties, ≥90th percentile for early preterm birth rate (N=230)**	
	Median Value	Median percentile	Median Value	Median percentile
**Overall SVI**	6.0	0.27	9.8	0.88
Component 1: Socioeconomic Status				
Overall (Component 1)	1.1	0.24	3.2	0.87
Residents living below poverty, %	10.9	0.24	23.3	0.88
Residents unemployed, %	4.4	0.31	8.0	0.83
< high school education, %	8.2	0.21	18.8	0.80
Median Income, $	30068	0.25	21334	0.84
Component 2: Household Composition and Disability				
Overall (Component 2)	1.6	0.20	2.4	0.79
Age ≥65 years, %	17.2	0.41	16.7	0.36
Age ≤17 years, %	21.7	0.40	23.1	0.60
Age ≥5 years with a disability, %	12.5	0.22	17.6	0.67
Single-parent household, %	6.9	0.28	11.0	0.86
Component 3: Minority Status and Language				
Overall (Component 3)	1.0	0.49	1.3	0.67
Minority, %	13.2	0.43	45.3	0.85
Speak English “Less than well”, %	1.0	0.58	0.6	0.42
Component 4: Housing and Transportation				
Overall (Component 4)	2.5	0.49	3.1	0.81
Multi-Unit structure, %	5.4	0.72	1.8	0.34
Mobile homes, %	6.4	0.30	21.5	0.82
Crowding, %	1.9	0.48	2.3	0.60
No Vehicle, %	5.2	0.41	8.8	0.85
Group Quarters, %	2.0	0.50	2.5	0.59

© 2023 Khan et al. *JAMA Network Open.*

eTable 3. Distribution of Percent Change in Age-Standardized and Age-Specific National and County-Level Preterm and Early Preterm Birth Rates From 2007 to 2019 in the US

	National % Change (95% CI)	Minimum, %	10th %, %	Median, %	90th %, %	Maximum, %
Preterm Birth (<37 weeks gestational age)						
Age-standardized	-5.0 (-10.7, 0.9)	-49.8	-16.4	-3.7	12.0	399.5
Age-specific						
15-19 years	-2.7 (-8.8, 3.9)	-55.4	-18.7	-3.4	13.7	451.9
20-24 years	-4.8 (-10.3, 1.1)	-55.7	-19.2	-4.9	12.7	418.9
25-29 years	-4.6 (-10.9, 2.1)	-53	-17.7	-4.7	11.4	365.9
30-34 years	-7.5 (-12.9, -1.8)	-44.5	-17.9	-5.1	9.2	312.0
35-39 years	-5.6 (-10.9, 0)	-36.8	-15.8	-3.9	10.4	271.7
40-44 years	-0.9 (-5, 3.3)	-22.4	-14.2	-1.3	15.5	32.7
Early Preterm (<34 weeks gestational age)						
Age-standardized	-3.0 (-6.4, 0.5)	-44.5	-16.7	-0.4	18.8	470.7
Age-specific						
15-19 years	-1.4 (-7.2, 4.8)	-42.2	-21.1	-2.2	18.0	442.4
20-24 years	-1.9 (-5.4, 1.6)	-49.9	-19.8	-1.7	20.6	512.4
25-29 years	-0.4 (-4.3, 3.8)	-46.2	-16.7	0.2	20.5	476.4
30-34 years	-5.8 (-8.9, -2.7)	-45.9	-19	-2.2	17.9	452.8
35-39 years	-6.7 (-9.5, -3.8)	-44.1	-22	-4.1	14.7	434.3
40-44 years	-4.9 (-9.3, -0.4)	-34	-24.5	-8.1	13.8	48.8

Positive percent change represents increase and negative percent change represents decrease in age-standardized rates of preterm and early preterm birth

© 2023 Khan et al. *JAMA Network Open.*

eTable 4. Age-Specific Rates of Preterm and Early Preterm Birth at the National and County-Level in the United States, 2019

National Rates (per 100 live births)				US County-Level Age-Specific Rates (per 100 live births)						
Age, years	Number of counties	Number of births	Rate (95% CI)	Min	10th %	Median	90th %	Max	90th-10th percentile (95% CI)	90th/10th percentile (95% CI)
Preterm Birth (<37 weeks gestational age)										
15-19	328	23971	14 (13.8, 14.1)	6.1	11.3	13.7	16.5	74.1	5.2 (4.6, 5.9)	1.5 (1.4, 1.5)
20-24	1191	85181	12.1 (12.0, 12.2)	5.7	9.4	11.6	14.9	65.6	5.5 (5.2, 5.8)	1.6 (1.5, 1.6)
25-29	1339	121433	11.3 (11.2, 11.3)	5.7	8.8	10.9	14.3	56.3	5.5 (5.1, 5.9)	1.6 (1.6, 1.7)
30-34	984	124263	11.4 (11.3, 11.5)	6.7	9.1	11.4	14.6	55.9	5.5 (5.2, 5.8)	1.6 (1.6, 1.6)
35-39	559	77584	13.5 (13.5, 13.6)	8.7	10.7	13.6	17.3	58.4	6.6 (5.8, 6.9)	1.6 (1.5, 1.7)
40-44	179	20067	16.7 (16.5, 16.9)	11	13.3	16.0	19.4	26	6.1 (5.2, 7)	1.5 (1.4, 1.5)
Early Preterm (<34 weeks gestational age)										
15-19		7797	4.5 (4.4, 4.6)	2.3	3.4	4.4	5.8	23.8	2.4 (2.2, 2.7)	1.7 (1.6, 1.8)
20-24	1187	25117	3.6 (3.5, 3.6)	1.9	2.6	3.4	4.6	22.6	2 (1.9, 2.2)	1.8 (1.7, 1.9)
25-29	1333	34659	3.2 (3.2, 3.2)	1.7	2.3	3.0	4.3	19.6	2 (1.8, 2.1)	1.9 (1.8, 1.9)
30-34	981	34588	3.2 (3.1, 3.2)	1.7	2.3	3.1	4.4	21.2	2.1 (1.8, 2.2)	1.9 (1.8, 2)
35-39	554	21777	3.8 (3.8, 3.9)	2.1	2.7	3.7	5.1	24.1	2.4 (2.2, 2.6)	1.9 (1.8, 2)
40-44	178	5829	4.9 (4.7, 5.0)	2.6	3.4	4.7	5.9	9.9	2.5 (2.1, 2.7)	1.7 (1.6, 1.8)

© 2023 Khan et al. *JAMA Network Open.*

eTable 5. National Age-Standardized Rates of Preterm Birth Outcomes and Distribution of Age-Standardized Rates of Preterm Birth Among US Counties in 2007 and 2019 among Individuals with a Singleton First Live Birth

	**National Rates (per 100 live births)**			**US County-Level Age-Standardized Rates (per 100 live births)**						
	Number of counties	Number of births	Age-standardized rate or change (95% CI)	Minimum	10th percentile	Median	90th percentile	Maximum	90th-10th percentile (95% CI)	90th/10th percentile ratio (95% CI)
Preterm Birth (<37 weeks gestational age)										
2007	1447	180821	10.5 (10.5, 10.6)	6.6	9.0	10.9	13.8	20.0	4.8 (4.5, 4.9)	1.5 (1.5, 1.5)
2019	1447	141029	10.2 (10.1, 10.2)	5.5	8.7	10.5	13.2	49.2	4.5 (4.4, 4.8)	1.5 (1.5, 1.6)
% Change	1447	--	-4.6 (-10.8, 2.1)	-42.5	-15.4	-4.1	9.6	339.1	–	–
Early Preterm (<34 weeks gestational age)										
2007	1446	53142	3.1 (3, 3.1)	1.5	2.5	3.2	4.2	7.0	1.7 (1.7, 1.9)	1.7 (1.7, 1.8)
2019	1446	41697	3 (3, 3)	1.8	2.4	3.1	4.3	14.3	1.9 (1.7, 1.9)	1.8 (1.7, 1.8)
% Change	1446	--	-1.9 (-5.7, 2.1)	-43.3	-14.9	-0.7	17	311.2	–	–

© 2023 Khan et al. *JAMA Network Open.*

eFigure 1. Age-Standardized Preterm Birth Rates in 2007 Among US States, Combined Statistical Areas, and Counties*

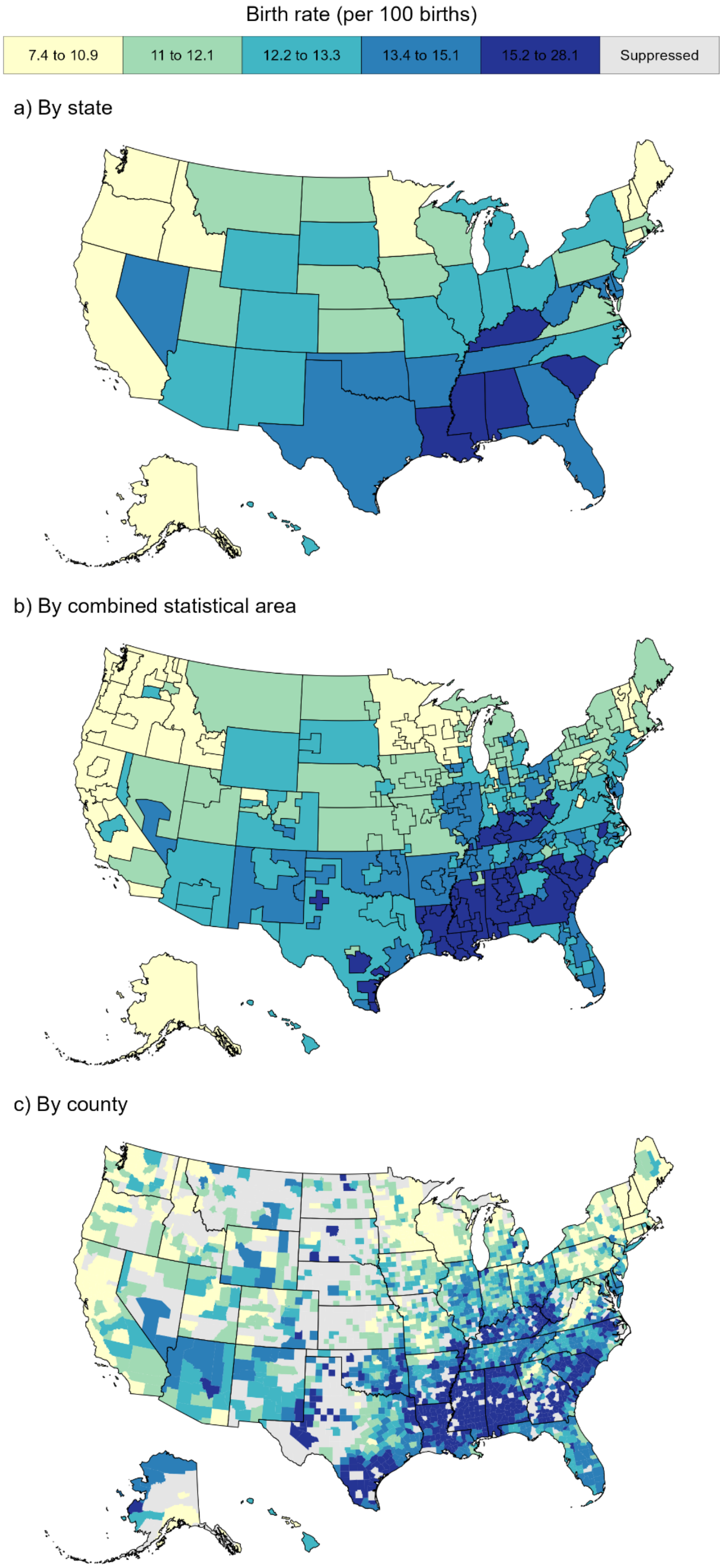

*For a county to be included on these maps, the estimated rates were required to be reliable (i.e., the credible interval width was less than the point estimate) and the total number of births was ≥100 for every year in the study period.

© 2023 Khan et al. *JAMA Network Open.*

eFigure 2. Age-Standardized Early Preterm Birth Rates in 2007 Among US States, Combined Statistical Areas, and Counties*

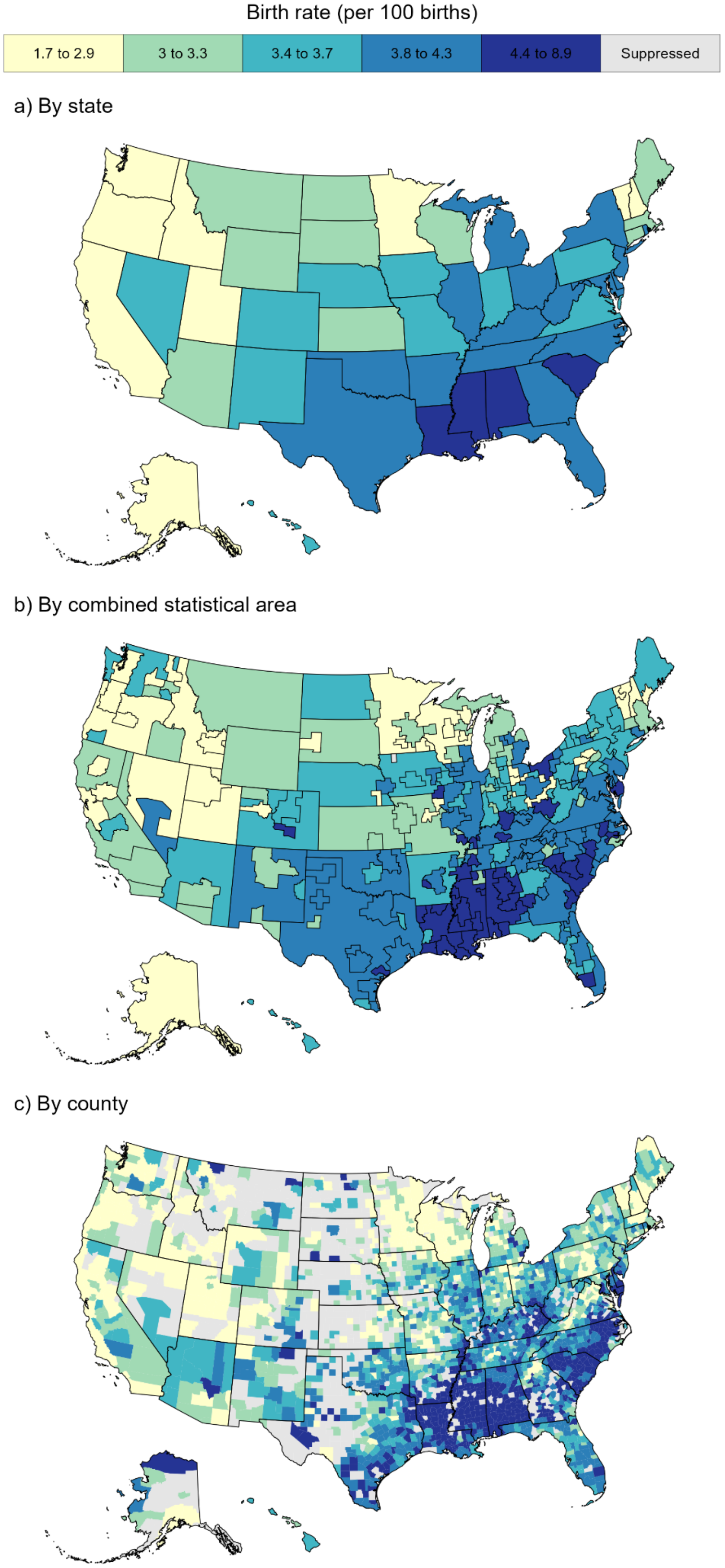

*For a county to be included on these maps, the estimated rates were required to be reliable (i.e., the credible interval width was less than the point estimate) and the total number of births was ≥100 for every year in the study period.

© 2023 Khan et al. *JAMA Network Open.*

eFigure 3. Age-Standardized Early Preterm Birth Rates in 2019 Among US States, Combined Statistical Areas, and Counties

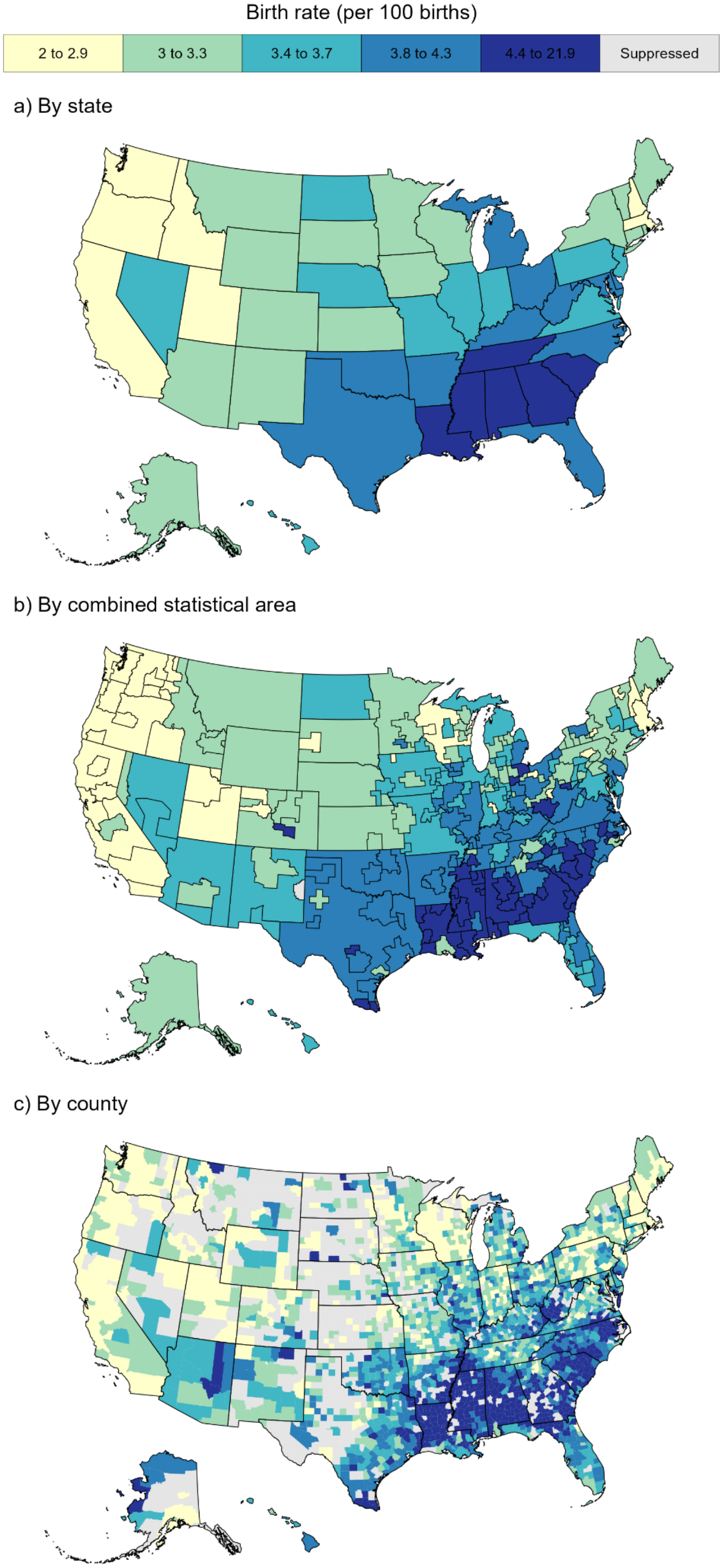

*For a county to be included on these maps, the estimated rates were required to be reliable (i.e., the credible interval width was less than the point estimate) and the total number of births was ≥100 for every year in the study period.

© 2023 Khan et al. *JAMA Network Open.*

eFigure 4. Total Percent Change in Age-Standardized Early Preterm Birth Rates Between 2007-2019 among US States, Combined Statistical Areas, and Counties*

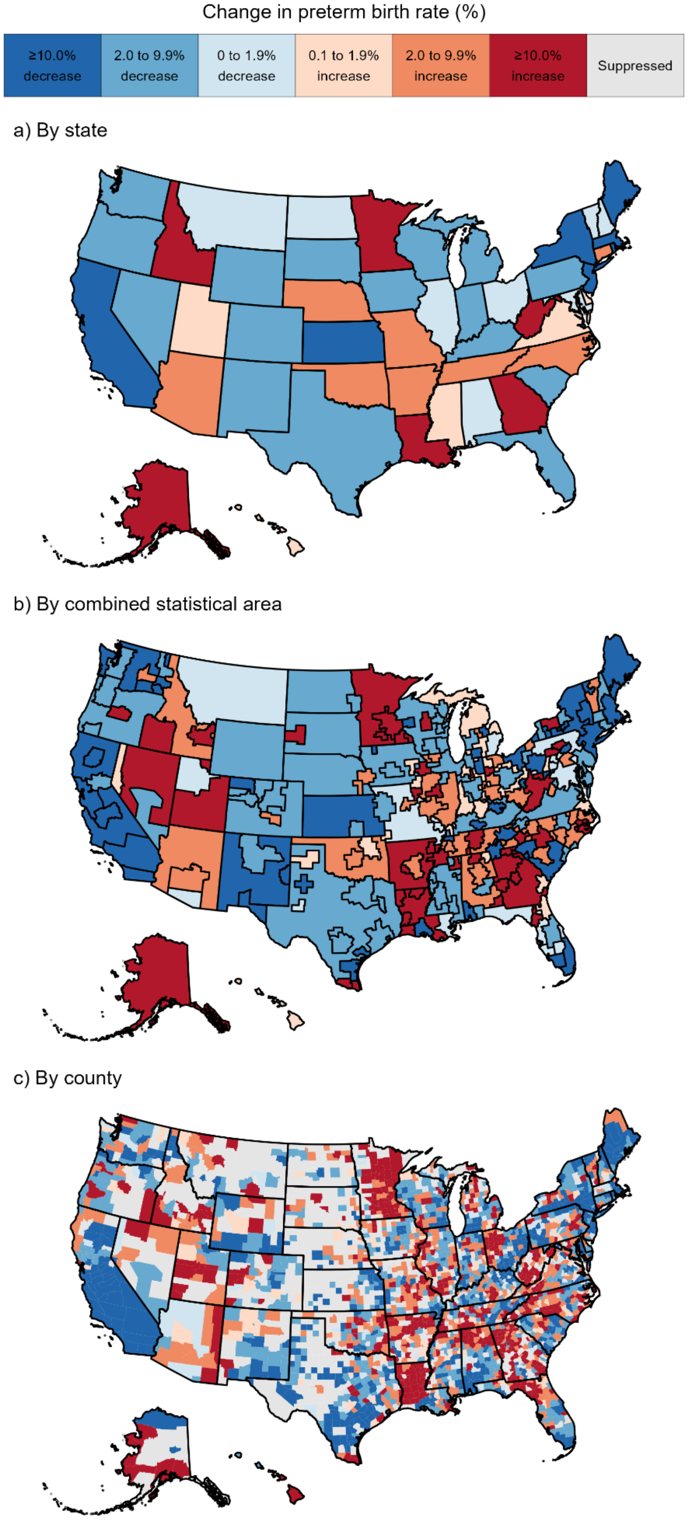

*Mapped values are the point estimates of percent change calculated using log-linear regression from estimated rates. For a county to be included on these maps, the estimated rates were required to be reliable (i.e., the credible interval width was less than the point estimate) and the total number of births was ≥100 for every year in the study period.

eFigure 5. Annual Rate Ratios (95% CI) for Preterm Birth Rate and Early Preterm Birth Rate Stratified by Quartiles of the County-Level Social Vulnerability Index, 2007-2019*

© 2023 Khan et al. *JAMA Network Open.*

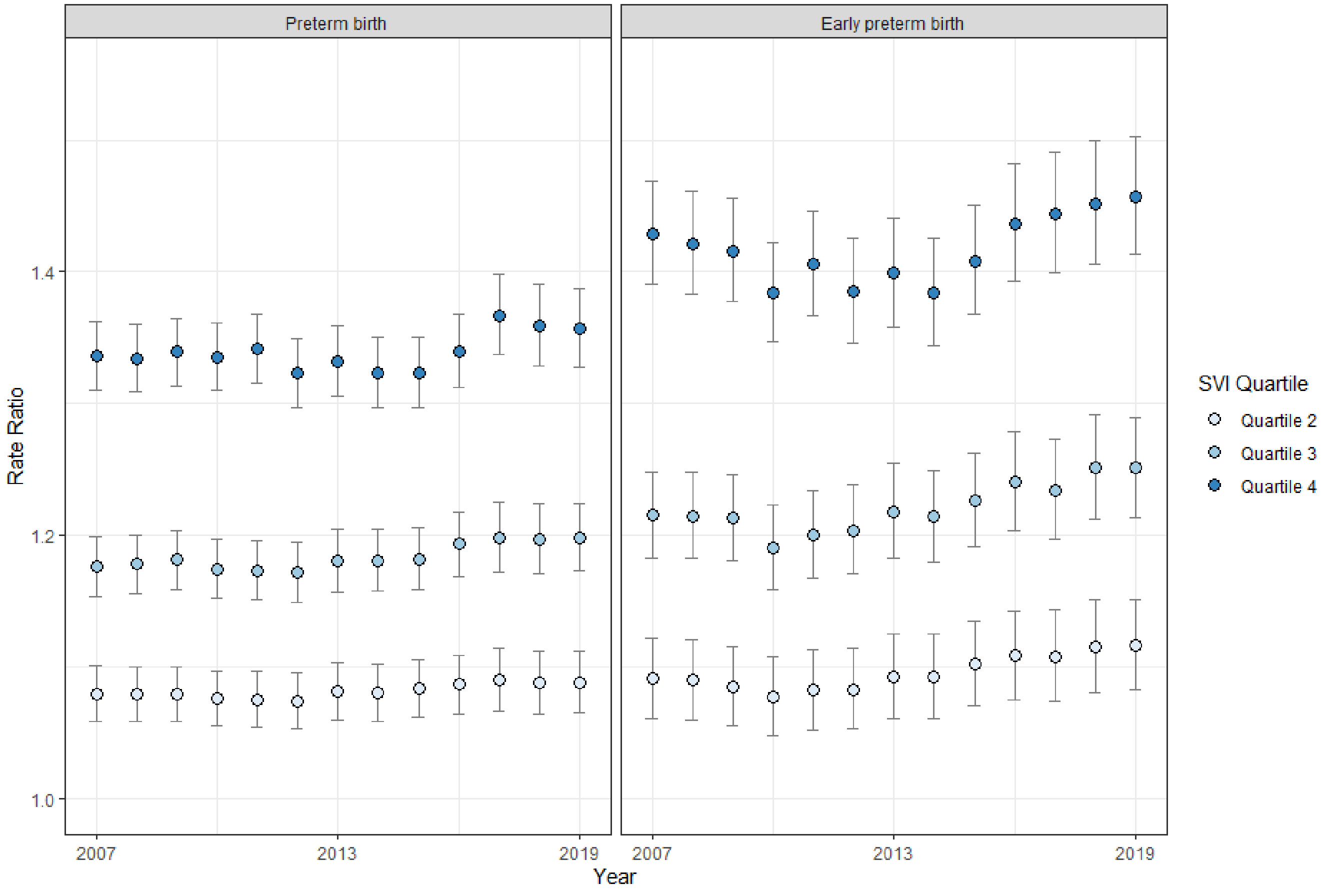

*We used the 2010 SVI for years 2007-2011, the 2014 SVI for years 2012-2015, the 2016 SVI for years 2016-2017, and the 2018 SVI for years 2018-2019
#The first quartile of SVI is the reference group.

© 2023 Khan et al. *JAMA Network Open.*

eFigure 6. Annual National and Distribution of County-Level Age-Standardized Preterm (A) and Early Preterm (B) Birth Rates
The bottom border, middle line, and the top border of the boxes represent the 25th, 50th, and 75th percentiles, respectively, across all counties; whiskers the full range across counties (excluding outliers); and circles, the national rate

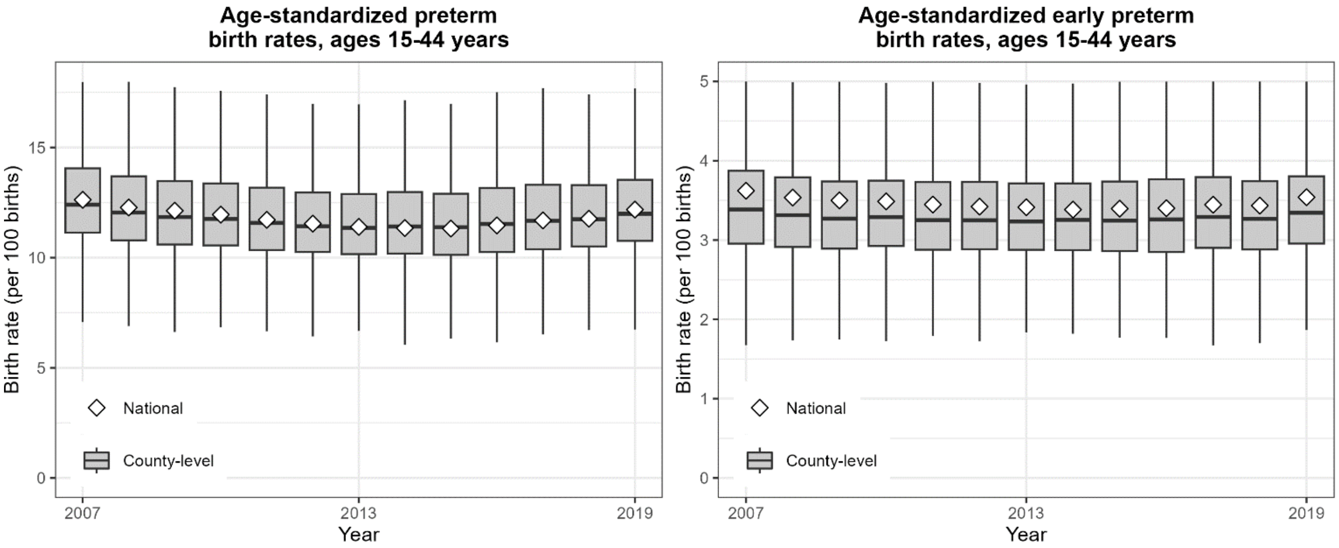

© 2023 Khan et al. *JAMA Network Open.*

eFigure 7. Distributions of Trends* Among US Counties for Early Preterm Birth Between 2007-2019 Overall and Stratified By 5-Year Age Groups

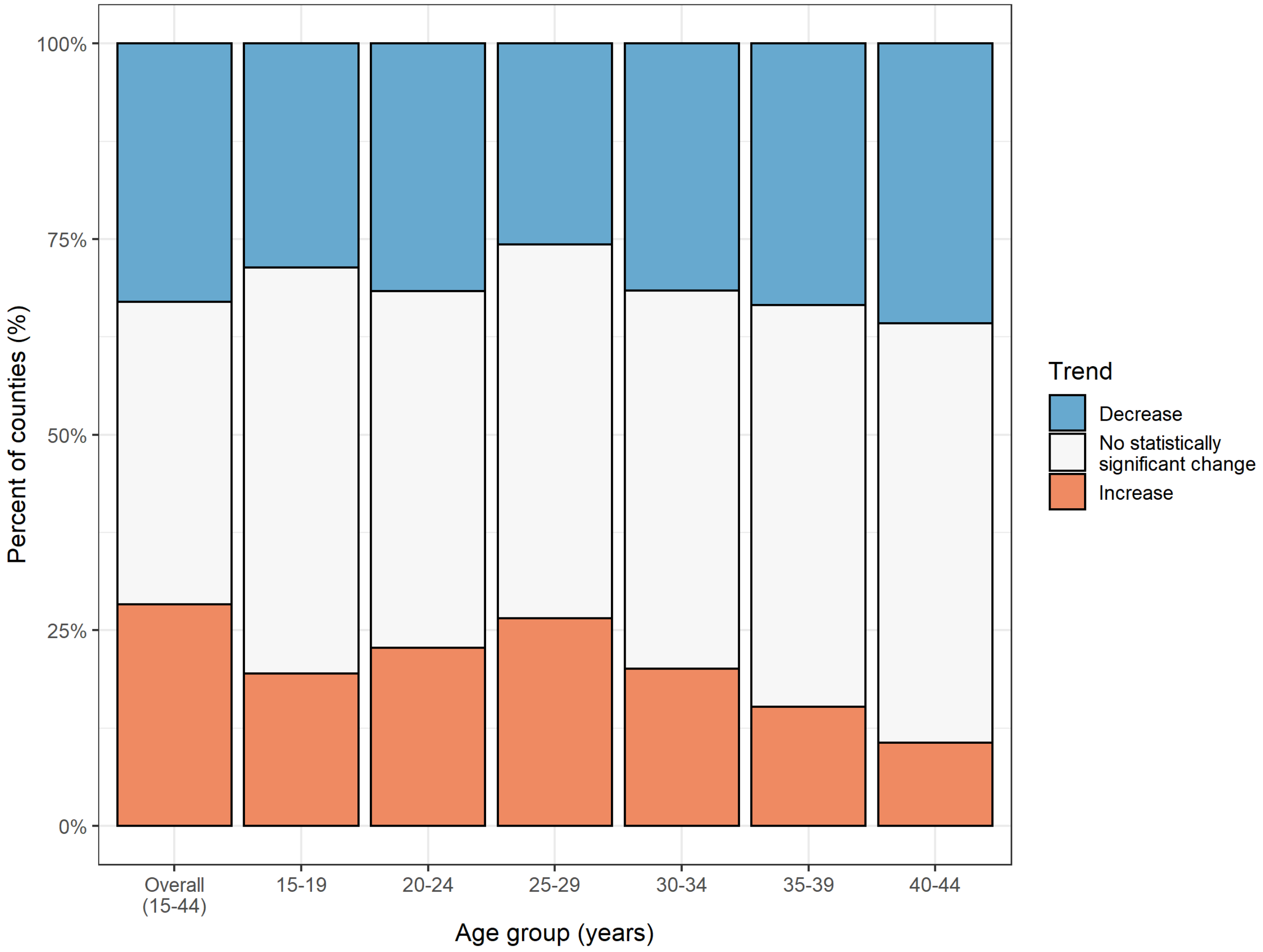

© 2023 Khan et al. *JAMA Network Open.*
