# Supplementary material for: US County–Level Variation in Preterm Birth Rates, 2007-2019
Source: JAMA Netw Open. 2023 Dec 8;6(12):e2346864. doi: 10.1001/jamanetworkopen.2023.46864 (PMC10709777; doi:10.1001/jamanetworkopen.2023.46864)
Supplement: Supplement 2. — Data Sharing Statement [file jamanetwopen-e2346864-s002.pdf]

Data Sharing Statement

Khan. US County–Level Variation in Preterm Birth Rates, 2007-2019. *JAMA Netw Open*. Published December 08, 2023. doi:10.1001/jamanetworkopen.2023.46864

Data
Data available: Yes
Data types: Deidentified participant data
How to access data: Deidentified data are available upon request through the CDC
When available: With publication

Supporting Documents
Document types: None

Additional Information
Who can access the data: Deidentified data are available upon request through the CDC
Types of analyses: Deidentified data are available upon request through the CDC
Mechanisms of data availability: Deidentified data are available upon request through the CDC
Any additional restrictions: Deidentified data are available upon request through the CDC with a signed data use agreement
